# Population Pharmacokinetic Modeling of Tribendimidine Metabolites in Opisthorchis viverrini-Infected Adults

**DOI:** 10.1128/AAC.00655-16

**Published:** 2016-09-23

**Authors:** Fiona Vanobberghen, Melissa A. Penny, Urs Duthaler, Peter Odermatt, Somphou Sayasone, Jennifer Keiser, Joel Tarning

**Affiliations:** a Swiss Tropical and Public Health Institute, Basel, Switzerland; b University of Basel, Basel, Switzerland; c National Institute of Public Health, Vientiane, Lao People's Democratic Republic; d Mahidol-Oxford Tropical Medicine Research Unit, Faculty of Tropical Medicine, Mahidol University, Bangkok, Thailand; e Centre for Tropical Medicine and Global Health, Nuffield Department of Medicine, University of Oxford, Oxford, United Kingdom

## Abstract

There is a pressing need for alternative treatments against the liver fluke Opisthorchis viverrini. Oral tribendimidine is a promising candidate, but its population pharmacokinetic properties are unknown. Two phase IIa trials were conducted in Laos in O. viverrini-infected adults receiving single oral doses of 25 to 600 mg tribendimidine administered as different formulations in each study (study 1 used 200-mg tablets, and study 2 used 50-mg tablets). Venous whole blood, plasma, and capillary dried blood spots were sampled frequently from 68 adults, and concentrations of the tribendimidine metabolites dADT (deacetylated amidantel) and adADT (acetylated dADT) were measured. Population pharmacokinetics were assessed by using nonlinear mixed-effects modeling. The relationship between drug exposure and cure (assessed at 21 days posttreatment) was evaluated by using univariable logistic regression. A six-transit compartment absorption model with a one-disposition compartment for each metabolite described the data well. Compared to the 50-mg formulation (study 2), the 200-mg formulation (study 1) had a 40.1% higher mean transit absorption time, a 113% higher dADT volume of distribution, and a 364% higher adADT volume of distribution. Each 10-year increase in age was associated with a 12.7% lower dADT clearance and a 21.2% lower adADT clearance. The highest cure rates (≥55%) were observed with doses of ≥100 mg. Higher dADT, but not adADT, peak concentrations and exposures were associated with cure (*P* = 0.004 and 0.003, respectively). For the first time, population pharmacokinetics of tribendimidine have been described. Known differences in the 200-mg versus 50-mg formulations were captured by covariate modeling. Further studies are needed to validate the structural model and confirm covariate relationships. (This study has been registered with the ISRCTN Registry under no. ISRCTN96948551.)

## INTRODUCTION

Helminthic infections present a challenging public health problem, with over 40 million people worldwide being infected with foodborne trematodes (1). The burden is disproportionately higher in resource-limited settings (2). In Southeast Asia, an estimated 8 million to 10 million people are infected with the liver fluke Opisthorchis viverrini (1). While most infections are symptom-free, severe manifestations may occur in the bile duct or gallbladder (cholangitis, cholecystitis, and cholestithiasis), and O. viverrini is a risk factor for the bile duct cancer cholangiocarcinoma (3). Praziquantel is the only drug currently available; therefore, there is a pressing need to identify alternatives (4, 5). Efforts are being made to develop new or to repurpose old drugs for helminthic treatment (2, 6).

Oral tribendimidine was first synthesized in China in the 1980s and has been marketed there since 2004 against hookworms, Ascaris lumbricoides, and Enterobius vermicularis (7). It is a promising candidate for the treatment of O. viverrini infection (8). In an open-label, randomized, exploratory, phase II trial, tribendimidine had high efficacy against O. viverrini, with no difference compared to praziquantel (9), and in a subsequent pair of phase IIa dose-finding trials, excellent efficacy at tribendimidine doses of 100 mg and above was observed, with the highest efficacy being observed at a dose of 400 mg (cure rate, 91.5%; egg reduction rate, 99.9%) (26). Tribendimidine is highly unstable and degrades spontaneously into deacetylated amidantel (dADT) and terephthalaldehyde (TPAL) in water without the involvement of metabolic enzymes (10). This process is accelerated at low pHs such as those in the gastrointestinal tract; therefore, the tablets are commercialized as a formulation with an enteric coating. dADT is partially converted to acetylated dADT (adADT), with 35% to 53% being excreted unchanged in urine (10, 11), and TPAL is metabolized completely into terephthalic acid (TPAC) (10). TPAL and TPAC are pharmacologically inactive metabolites, whereas dADT is highly active, and adADT has marginal or no anthelminthic activity (12).

Knowledge of drug pharmacokinetic (PK) properties is essential to inform dosing and may inform drivers of cure, yet to date, limited data are available to inform drug exposure to oral doses of tribendimidine. Small studies in China (each with ≤30 participants) have described the basic PK properties in healthy volunteers (10, 11, 13–15). In the phase IIa dose-finding trials mentioned above, we investigated the model-independent PK properties among persons infected with O. viverrini (27). However, the population PK properties, which are crucial in order to assess influential covariates and to develop a rational framework for future clinical trial simulations, remain unknown. The aim of this study was to determine the population PK properties of tribendimidine in persons infected with O. viverrini and to investigate the relationship with cure.

## MATERIALS AND METHODS

### Study design and ethical considerations.

Two phase IIa, open-label, randomized, ascending-dose-finding trials were conducted in Laos in November 2012 and October 2013, with similar methodologies. Details on the trials have been presented elsewhere (26). Eligible patients identified through clinical examination and interviews were those who did not suffer from major systemic or chronic illness and psychiatric disorders and were not pregnant. Stool samples were taken prior to treatment and 21 days later to estimate the egg burden before and after treatment in order to quantify the pharmacodynamic (PD) effects of tribendimidine against O. viverrini. At each of these time points, two stool samples were collected on different days within a maximum of 3 days, and two Kato-Katz thick smears (41.7 mg) were prepared from each stool specimen (26). In the framework of these trials, O. viverrini-positive patients were admitted to Champasack Provincial Hospital, Pakse, Laos, for 24 h for participation in a PK study. The PK study, including noncompartmental PK results, has been reported in full elsewhere (27), and details are given below.

The studies were approved by the Ethics Committee of the Ministry of Health, Vientiane, Laos (reference no. 009/NECHR); the Ethical Committee of the Canton of Basel-Stadt and Basel-Land, Basel, Switzerland (EKBB) (reference no. 375/11); and the Liverpool School of Tropical Medicine Research Ethics Committee, United Kingdom (reference no. 12.02RS). This study was registered with the ISRCTN Registry (no. ISRCTN96948551). Informed consent written in Lao language was read and explained by a researcher to each participant, and all participants provided written informed consent.

### Treatment and blood sampling.

Participants received single oral doses of 200, 400, and 600 mg tribendimidine (using 200-mg tablets with enteric coating) in the first trial and doses of 25, 50, 100, and 200 mg (using 50-mg tablets with enteric coating) in the second trial (all tablets were produced by Shandong Xinghua Pharmaceutical Corporation, China). Of note, different absorption properties of the two formulations were hypothesized since they were observed previously (27).

Venous blood sampling was performed at ∼0, 1, 2, 3, 4, 4.5, 5, 6, 8, 10, and 24 h postdose to assess both whole-blood and plasma drug concentrations. As detailed elsewhere (27), 4 ml venous blood was collected, 1 ml was transferred to a labeled tube within 30 min postsampling, and the remaining sample was centrifuged to produce plasma. The samples were stored at −80°C. Capillary blood samples (0.1 ml) were taken by fingertip puncture at ∼0, 2, 4, 5, 8, and 24 h postdose to validate a novel dried blood spot (DBS) method for drug quantification (16). Four drops of blood were transferred onto filter paper and dried for ∼1 h. Plasma and DBS measurements were performed in both studies, while whole-blood measurements were performed in the first study only.

### Drug measurements.

The concentrations of the active metabolites dADT and adADT in venous whole blood, plasma, and capillary blood on filter paper were quantified by using a validated liquid chromatography-tandem mass spectrometry method (16). The lower limit of quantification (LLOQ) was 1 ng/ml for whole blood and plasma, and the LLOQ was 10 ng/ml for DBS in the first study (16); in the second study, the LLOQ for DBS was reduced to 1 ng/ml since the doses were up to 10 times lower. The within-day and between-day accuracy and precision at low-, mid-, and high-quality-control levels were below 15% (LLOQ, 20%) throughout the analysis of clinical samples (27).

### Pharmacokinetic and statistical analyses.

Data were processed by using Stata version 12 (StataCorp, College Station, TX). Graphics were created by using R (version 3.0.2; R Foundation for Statistical Computing).

Whole-blood, plasma, and DBS concentrations were pooled across both studies, and molar units of dADT and adADT were transformed into their natural logarithms and modeled simultaneously by using nonlinear mixed-effects modeling. Estimations and simulations were performed by using NONMEM 7.1.2 (17) with Piraña 2.8.2 (Piraña Software and Consulting) and Perl-speaks-NONMEM (PsN) (18). The first-order conditional estimation method with interactions or the Laplacian estimation method was used throughout modeling. A fixed renal clearance value for dADT of 35% was assumed since no urine data were available (10); the remaining 65% was assumed to be completely metabolized into adADT. One-compartment and two-compartment disposition models were considered for each metabolite, using a first-order absorption model. Different absorption models were then evaluated by using the best-performing disposition model, i.e., first-order absorption and a more flexible transit absorption model (stepwise addition of transit compartments with the transit rate constant set equal to the absorption rate constant). Relative bioavailability (*F*), fixed to unity for the population but allowing for interindividual variability on this parameter, was evaluated. Interindividual random variability in all parameters was modeled exponentially by using the formula θ_*i*_ = θ_*TV*_ × exp(η_*i*__,__θ_), where θ_*i*_ is the individually estimated parameter value for the *i*th patient, θ_*TV*_ is the typical parameter value for the modeled population, and η_*i*__,__θ_ is interindividual random variability, assumed to be normally distributed with zero mean and variance ω^2^. Potential correlations between the clearance and volume parameters for both metabolites were evaluated with a full variance-covariance matrix. The residual unexplained variability was modeled as a separate additive error for each metabolite on the log-transformed concentrations, which is essentially equivalent to an exponential residual error on the arithmetic scale. A proportional transformation factor for plasma, DBS, and whole-blood samples (without interindividual variability) was evaluated to allow for systematic differences between sampling matrices (19). Values below the limit of quantification were omitted initially but subsequently evaluated as censored observations using the M3 method (20).

Body weight was evaluated as an allometric function on all clearance and volume parameters (i.e., exponents of 0.75 and 1 for clearance and volume parameters, respectively [21]). Other biologically plausible covariates (age, sex, creatinine clearance, formulation, and dose) were evaluated by using a stepwise inclusion (*P* < 0.05) and elimination (*P* > 0.01) approach, with linear functions for the continuous variables (namely, age, creatinine clearance, and dose, centered on the median value). Creatinine clearance was calculated by using the Chronic Kidney Disease Epidemiology Collaboration equation (22). We also considered estimating an interaction between the mean transit time and whether the dose contained split tablets (25-mg dose only) or not.

Model discrimination was performed by assessing changes in the objective function value (ΔOFV) (calculated as proportional to minus twice the log likelihood of data) and associated χ^2^ tests. Goodness of fit was assessed by inspection of diagnostic plots with consideration of parameter estimate (eta) shrinkage and epsilon shrinkage (23). A prediction-corrected visual predictive check (VPC) was performed for the final model (*n* = 1,000, stratified on metabolite). DBS samples from study 1 were omitted from the VPC due to the 10-fold difference in the LLOQ. The VPC was visualized by plotting the 5th, 50th, and 95th percentiles of the observed data overlaid with the 95% confidence intervals of the same percentiles of the simulated data. The simulated and observed fractions of data below the limit of quantification were also visualized to evaluate the impact of data censoring. For the final model, relative standard errors and 95% confidence intervals of parameter estimates were derived by bootstrap diagnostics stratified by formulation (104 replications only, due to long run times).

Individually estimated secondary PK parameters, including maximum concentration of drug (*C*_max_), time to maximum concentration of drug (*T*_max_), half-life, and area under the concentration-time curve (AUC) from 0 to 72 h postdose, were derived directly by NONMEM for each metabolite. In addition, a regression analysis of the terminal phase of model-predicted individual concentration-time profiles was performed to obtain estimates of half-life.

O. viverrini egg burdens, expressed as eggs per gram of stool (epg), were determined as the means for the four counts (or as many as were available) from the two slides of the two stool samples multiplied by 24. The relative reduction in egg burden was calculated for each participant as 100 × (burden at enrollment − burden at 21 days)/(burden at enrollment). Cure was defined as no detection of eggs at 21 days. Univariable logistic regression was used to assess the relationship between individually estimated exposure parameters (i.e., *C*_max_ and AUC) and cure.

## RESULTS

Overall, 68 participants were enrolled: 31 in the first study (13, 9, and 9 participants received 200, 400, and 600 mg, respectively) and 37 in the second study (9, 9, 9, and 10 participants received 25, 50, 100, and 200 mg, respectively). Thirty-five participants (51%) were female, and the median age was 42 years (interquartile range, 32 to 47 years), the median weight was 52 kg (47 to 57 kg), the median creatinine clearance was 66 ml/min per 1.73 m^2^ (50 to 112 ml/min per 1.73 m^2^), and the median O. viverrini egg burden was 897 epg (437 to 1,817 epg). A total of 1,307 samples were analyzed for dADT (1,303 for adADT), of which 300 were whole-blood, 669 were plasma, and 338 were DBS samples. The mean number of whole-blood and plasma samples was 10 per participant, and that for DBS was 5 (plus a baseline sample for each participant). Overall, 15 (5%) whole-blood, 81 (12%) plasma, and 41 (12%) DBS dADT measurements were below the quantification limit; the corresponding figures for adADT were 38 (13%), 117 (18%), and 82 (24%), respectively.

### Structural model.

A one-compartment disposition model for each metabolite described the observed data well, with no further improvement with additional distribution compartments. The transit compartment absorption model was superior to a first-order absorption model, with an optimum number of 6 transit compartments. The addition of proportional transformation factors for both plasma and DBS versus blood did not improve the model significantly, nor did incorporation of interindividual variability for the relative bioavailability, and therefore, these models were not carried forward. Incorporating correlations between the clearance and volume parameters for both metabolites improved the model fit substantially (ΔOFV = −28); therefore, this model was carried forward. Omission of concentrations below the LLOQ resulted in model misspecification of the fraction of censored data (data not shown), which was described adequately by using the M3 method; therefore, the M3 method was used for the final model.

### Covariate modeling.

**FIG 1:**
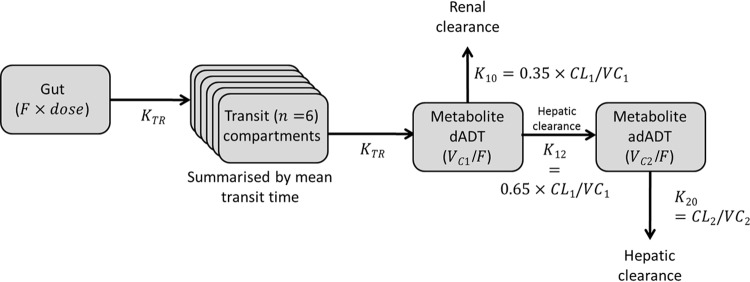
Final structural pharmacokinetic model for tribendimidine metabolites in adults with Opisthorchis viverrini infection. The model is a six-transit absorption model with a one-compartment disposition model for each metabolite, dADT and adADT. *F*, bioavailability; *K_TR_*, transit absorption rate; *n*, total number of compartments [mean transit time = (*n* + 1)/*K_TR_*]; CL, clearance; *V_C_*, apparent central volume of distribution (subscript 1 for dADT and subscript 2 for adADT).

A number of parameter-covariate relationships were significant in the stepwise covariate approach. Compared to the 50-mg formulation used in study 2, the 200-mg formulation used in study 1 had a 40.1% higher mean transit absorption time, a 113% higher dADT volume of distribution, and a 364% higher adADT volume of distribution. Each 10-year increase in age was associated with a 12.7% lower dADT clearance and a 21.2% lower adADT clearance. The model incorporating an interaction between the mean transit time and whether the dose used split tablets (25-mg dose only) or not did not converge. The final model and parameter estimates are shown in Fig. 1 and Table 1 (see File S1 in the supplemental material for NONMEM code).

**TABLE 1:** Population pharmacokinetic parameter estimates from the final model describing tribendimidine metabolites in adults infected with Opisthorchis viverrini^*a*^

Parameter	Population estimate^*b*^	95% confidence interval^*c*^	Relative SE^*c*^
No. of transit compartments	6 (fixed)	NA	NA
Mean transit time (h)	3.38	2.51, 4.63	14.5
Metabolite dADT			
CL/*F* (liters/h)	16.7	14.6, 18.1	5.12
*V_C_*/*F* (liters)	93.3	74.3, 112	8.49
σ (% CV)^*d*^	116	94.1, 141	12.6
Metabolite adADT			
CL/*F* (liters/h)	41.8	31.9, 55.2	13.5
*V_C_*/*F* (liters)	11.5	6.78, 20.3	25.0
σ (% CV)^*d*^	63.9	50.1, 77.3	15.6
Covariate effects (%)			
Formulation on mean transit time^*e*^	40.1	2.96, 96.9	48.7
Formulation on dADT *V_C_*/*F*^*e*^	113	53.0, 196	31.6
Formulation on adADT *V_C_*/*F*^*e*^	364	141, 499	29.0
Age on dADT CL/*F*, per 10 yr older	−12.7	−19.9, −8.08	22.5
Age on adADT CL/*F*, per 10 yr older	−21.2	−42.1, −3.72	43.4
Interindividual variability (% CV)^*d*^			
Mean transit time	88.5	72.0, 111	16.9
dADT CL/*F*	24.8	14.1, 34.6	34.9
dADT *V_C_*/*F*	101	62.5, 141	29.5
adADT CL/*F*	106	81.5, 150	19.8
adADT *V_C_*/*F*	134	82.9, 258	33.6
Correlations (% CV)^*f*^			
dADT CL/*F* and dADT *V_C_*/*F*	91.8	74.8, 98.9	26.6
dADT CL/*F* and adADT CL/*F*	−62.6	−92.9, −20.1	38.5
dADT CL/*F* and adADT *V_C_*/*F*	15.2	−49.6, 59.2	126
dADT *V_C_*/*F* and adADT CL/*F*	−46.1	−72.4, −9.37	53.3
dADT *V_C_*/*F* and adADT *V_C_*/*F*	51.8	−9.50, 72.8	37.3
adADT CL/*F* and adADT *V_C_*/*F*	34.0	−8.01, 68.9	54.3

a CL, clearance; *F*, bioavailability; *V_C_*, apparent central volume of distribution; σ, additive residual error; CV, coefficient of variation. Results shown are for a typical patient aged 52 years, weighing 51.5 kg, and receiving 50-mg tablets (study 2).

b Population estimates are from NONMEM.

c Confidence intervals and relative standard errors (SE) were estimated by bootstrap analysis (104 replications). NA, not applicable.

d Calculated as $\sqrt{\left(\exp(\eta_{\theta })-1\right)}\times 100$.

e Two-hundred-milligram tablets (study 1) versus 50-mg tablets (study 2).

f Calculated as $correlation\;estimates/\sqrt{\left(\eta_{\theta 1}\times \eta_{\theta 2}\right)}\times 100$.

### Model diagnostics.

**FIG 2:**
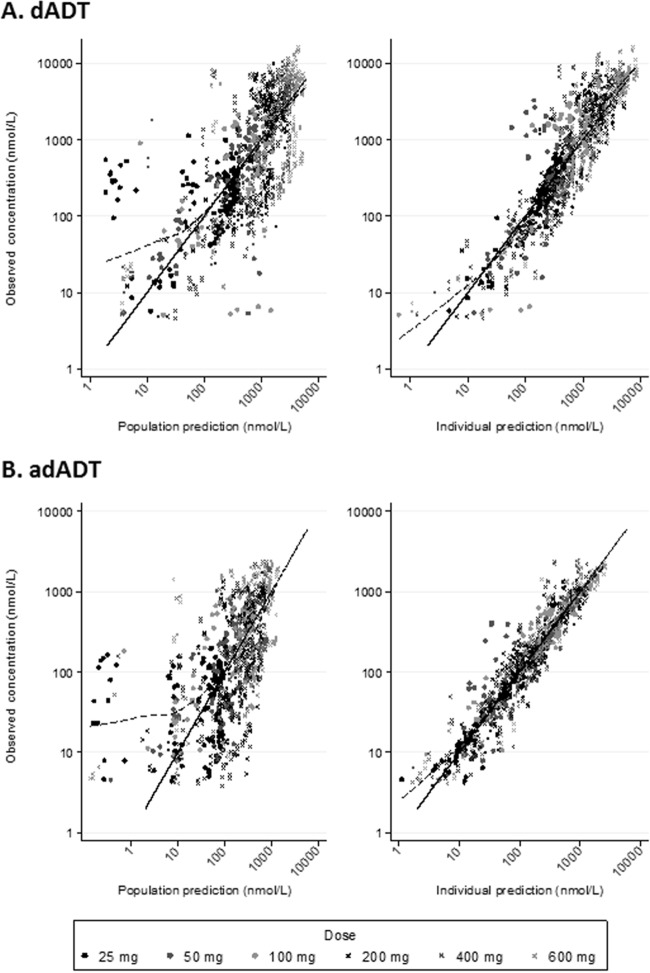
Goodness-of-fit diagnostics for the final pharmacokinetic model. From left to right, plots show observed versus population-predicted concentrations and observed versus individual predicted concentrations. Data points are shown by dose. Solid lines show the line of identity; dashed lines show a locally weighted regression line.

**FIG 3:**
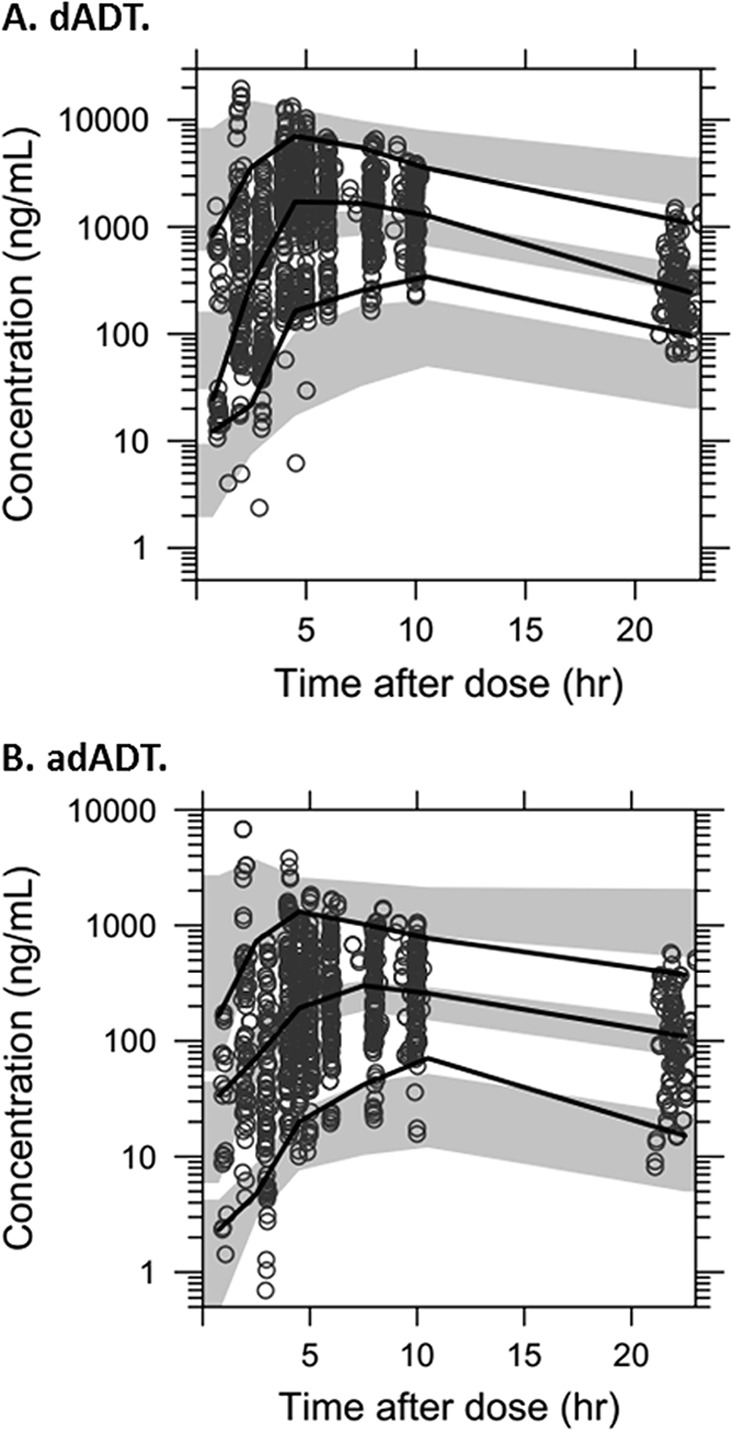
Visual predictive checks of the final pharmacokinetic model. Visual predictive checks illustrate the concentration of the metabolite (dADT [A] and adADT [B]) versus time. Open circles indicate the observed data; black lines indicate the 5th, 50th, and 95th percentiles of the observed data; and the gray bands indicate the 95% confidence intervals of the same percentiles of the simulated data. Results exclude DBS from study 1 due to the 10-fold different limit of quantification.

Goodness-of-fit diagnostics showed no obvious model misspecification, but a small deviation at low concentrations was noted, likely due to censoring of data below the limit of quantification (Fig. 2). For both metabolites, the population predictions tended to be underestimated for the lowest dose of 25 mg (Fig. 2A and B, left panels). Eta and epsilon shrinkages were low (≤12%). The prediction-corrected visual predictive checks suggested a reasonable model fit albeit with some misspecification for the 95th percentiles at 24 h and the 5th percentile for dADT (Fig. 3).

### Secondary PK parameters and outcomes.

Model-derived estimates [i.e., ln(2) × *V_C_*/CL] for the adADT elimination half-life were a factor of 10 lower than noncompartmental analysis (NCA) estimates (27). A regression analysis of the terminal phase of model-predicted individual concentration-time profiles showed almost identical terminal elimination half-lives between the two metabolites (Table 2), indicating that the adADT metabolite is subject to formation-rate-limited elimination. Therefore, the half-lives determined by regression should be used for true representation of the elimination half-life and are presented henceforth.

**TABLE 2:** Secondary pharmacokinetic parameter estimates from the final population pharmacokinetic model^*a*^

Metabolite and dose (mg)	Median *C*_max_ (ng/ml) (interquartile range)	Median *T*_max_ (h) (interquartile range)	Median half-life (h) (interquartile range)^*b*^	Median AUC (h · ng/ml) (interquartile range)
dADT				
25	67 (61–70)	1.75 (1.56–2.21)	4.67 (3.70–5.00)	488 (448–515)
50	105 (71–115)	12.20 (6.07–14.10)	2.81 (2.26–3.58)	957 (857–1,099)
100	246 (201–275)	5.99 (3.92–8.16)	3.30 (2.51–3.67)	2,275 (1,798–2,491)
200	414 (341–511)	7.76 (5.54–9.31)	4.09 (3.17–4.61)	3,924 (3,459–5,327)
400	821 (317–873)	7.07 (4.21–8.48)	4.83 (4.36–12.41)	7,798 (6,653–10,219)
600	953 (440–1,058)	6.54 (4.97–8.52)	5.00 (4.24–10.64)	10,831 (8,162–14,584)
adADT				
25	25 (5–34)	2.28 (2.06–2.43)	4.67 (3.70–5.00)	161 (44–235)
50	18 (16–42)	12.20 (6.18–14.20)	2.81 (2.26–3.58)	363 (199–398)
100	101 (91–109)	6.12 (4.42–8.47)	3.30 (2.51–3.67)	809 (690–1,013)
200	60 (38–237)	8.51 (6.04–9.79)	4.09 (3.17–4.61)	972 (436–2,399)
400	116 (69–202)	9.22 (7.96–11.20)	4.83 (4.36–12.41)	2,049 (878–4,975)
600	235 (215–387)	8.89 (5.52–11.90)	5.00 (4.24–10.63)	4,033 (2,958–6,050)

a *C*_max_, maximum concentration; *T*_max_, time to maximum concentration; AUC, area under the concentration-time curve (0 to 72 h).

b Estimated by regression analysis of the terminal phase of model-predicted individual concentration-time profiles.

As expected, the estimated *C*_max_, AUC, and elimination half-life values for dADT and adADT typically increased with higher doses, suggesting dose-proportional pharmacokinetics (Table 2). There was large variability in the estimated *T*_max_, except for the 25-mg dose, where split tablets were used and therefore the enteric coating of the tablet was destroyed.

**FIG 4:**
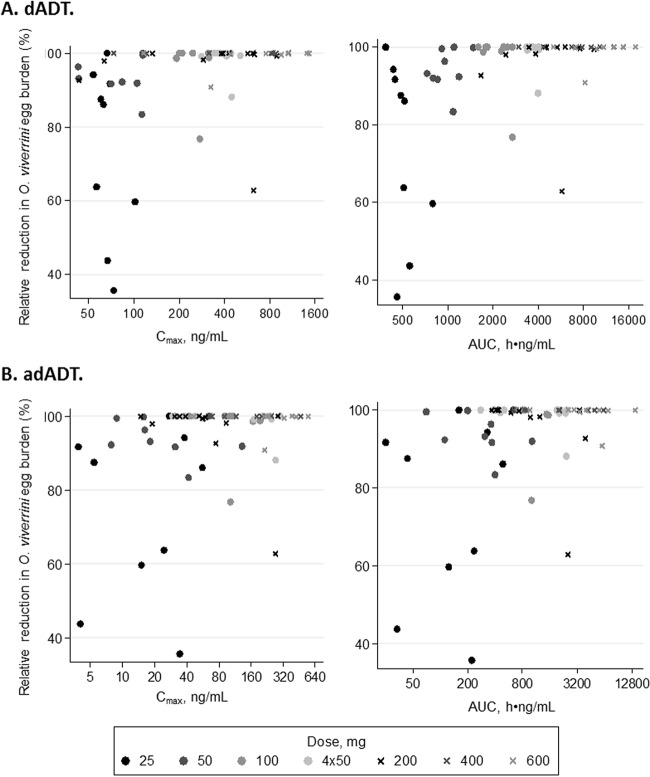
Relative reduction in O. viverrini egg burden by exposure parameter. Shown are observed relative reductions in O. viverrini egg burden from baseline to 21 days later versus estimated exposure parameters (*C*_max_ and AUC) for each patient, where a 100% relative reduction indicates cure. (A) Results for the metabolite dADT; (B) results for the metabolite adADT. Doses of 25, 50, and 100 mg and four 50-mg doses using 50-mg tablets in study 2 are indicated by filled circles with darker colors for lower doses, and doses of 200, 400, and 600 mg using 200-mg tablets in study 1 are indicated by crosses with darker colors for lower doses.

Among 67 patients with results at 21 days, cure rates ranged between 11% (25 mg) and 100% (400 mg). The median relative reductions in egg burden (interquartile ranges; ranges) were 86% (60 to 92%; 36 to 100%), 93% (92 to 100%; 83 to 100%), 100% (99 to 100%; 77 to 100%), 100% (99 to 100%; 63 to 100%), 100% (100 to 100%; 100 to 100%), and 100% (100 to 100%; 91 to 100%) for 25 mg, 50 mg, 100 mg, 200 mg, 400 mg, and 600 mg, respectively (Fig. 4). Cure was associated with higher dADT *C*_max_ and AUC values (*P* = 0.004 and 0.003, respectively). adADT exposure was not associated with cure (*P* ≥ 0.15).

## DISCUSSION

This first report on the population PK of tribendimidine, in persons infected with O. viverrini, supports the clinical development of tribendimidine for the treatment of liver fluke infections. We have shown that a central metabolite compartment model describes the PK of tribendimidine well, in agreement with data from previous analyses of healthy volunteers (10, 13, 15), with absorption being captured by a flexible-transit compartment model.

The availability of a relatively large sample size and intensive sampling were key strengths of this analysis, which combined data across two similar studies and a broad range of doses. However, this added a complication to the analysis, as there are known differences between the formulations of the tablets used in the two studies (200-mg tablets in the first study and 50-mg tablets in the second study), with the 200-mg tablets being more likely to “float” *in vitro* and hence delay absorption (27). These differences were captured by covariate modeling, with an interaction between formulation and mean transit absorption time (40% longer for the 200-mg tablets than for the 50-mg tablets). Furthermore, modeling indicated that the volume of distribution for both dADT and adADT was higher with the 200-mg than with the 50-mg formulation, but the reasons for this are not clear. However, the overall dADT exposures were similar between formulations and suggest that the effect of the formulation might have a limited clinical impact. Of note, the 200-mg tablet is that which is currently licensed for use in China. Besides formulation effects, interactions for age were incorporated into the model, with older age being associated with lower clearance for both metabolites, as might be expected (24). This could potentially result in underexposure in younger patients after standard dosing, and further studies are warranted to address dose optimization in this group of patients.

A further strength of this study was the availability of drug concentrations measured in three biological fluids, namely, whole blood, plasma, and DBS, and we were able to model these jointly, with no evidence of a difference between the fluids. Consistent with data from previous analyses (16, 27), our findings support the use of the novel DBS method for future PK studies, offering cheaper and more convenient sampling. The structural modeling applied in this study was used to inform the optimal design of a population PK phase IIb study using DBS with sparse sampling. The phase IIb study used a dose of 400 mg with 200-mg tablets. Subsequent work will aim to validate our model using the data from the phase IIb study.

We observed high correlation between clearance and volume of distribution for each of the individual metabolites, as we might expect given that treatment was administered orally. The interindividual variability in relative bioavailability was close to zero, suggesting that potential between-patient variability was fully explained by the implemented variance-covariance matrix. We assumed a fixed renal clearance of dADT of 35% based on data from a study from 2010 (10). An earlier study indicated that the renal clearance rate may vary between 35 and 58%, but it was not possible to examine this analysis in detail (11). Urine data were not available for the present study, so this could not be evaluated with our data. The fixed renal clearance value therefore simply acts as a scaling factor for the pharmacokinetic parameters.

Compared to a recent NCA of these data, our estimates of *C*_max_ and AUC were broadly comparable for the metabolite adADT but were somewhat lower for dADT (Table 3) (27). However, an NCA is highly dependent on the sampling design, and model-derived exposures might better correspond to the expected exposures. As discussed above, we observed that the metabolite adADT is subject to formation-rate-limited elimination, and regression of the terminal phase of individual concentration-time data should be used to obtain a true representation of the half-life; our estimates were similar to those determined by NCA.

**TABLE 3:** Comparison of our compartmental model results with data from NCA and the literature^*a*^

Metabolite, dose, and source	Participants	No. of participants	Median *C*_max_ (ng/ml) (interquartile range)	Median *T*_max_ (h) (interquartile range)	Median half-life (h) (interquartile range)^*b*^	Median AUC (h · ng/ml) (interquartile range)^*c*^
Metabolite dADT						
25 mg						
Compartmental model results	O. viverrini-infected individuals	9	67 (61–70)	1.75 (1.56–2.21)	4.67 (3.70–5.00)	488 (448–515)
NCA results^*d*^	O. viverrini-infected individuals	9	68 (62–88)	3 (3–4)	4 (4–5)^*e*^	485 (410–535)^*f*^
50 mg						
Compartmental model results	O. viverrini-infected individuals	9	105 (71–115)	12.20 (6.07–14.10)	2.81 (2.26–3.58)	957 (857–1,099)
NCA results^*d*^	O. viverrini-infected individuals	9	252 (230–327)	6 (4–8)	3 (3–4)^*e*^	1,249 (1,165–1,435)^*f*^
100 mg						
Compartmental model results	O. viverrini-infected individuals	9	246 (201–275)	5.99 (3.92–8.16)	3.30 (2.51–3.67)	2,275 (1,798–2,491)
NCA results^*d*^	O. viverrini-infected individuals	9	508 (372–566)	4 (3–5)	4 (3–5)^*e*^	2,475 (2,111–2,749)^*f*^
200 mg						
Compartmental model results	O. viverrini-infected individuals	23	414 (341–511)	7.76 (5.54–9.31)	4.09 (3.17–4.61)	3,924 (3,459–5,327)
NCA results for 4 50-mg tablets^*d*^	O. viverrini-infected individuals	10	701 (543–789)	4 (3–5)	4 (4–5)^*e*^	4,914 (4,184–5,640)^*f*^
NCA results for 1 200-mg tablet^*d*^	O. viverrini-infected individuals	13	744 (562–1,098)	5 (4–8)	4 (3–5)^*e*^	6,459 (5,658–7,802)^*f*^
Yuan et al.^*g*^^,^^*h*^	Healthy volunteers	12	370	3.60	4.25	2,120
400 mg						
Compartmental model results	O. viverrini-infected individuals	9	821 (317–873)	7.07 (4.21–8.48)	4.83 (4.36–12.41)	7,798 (6,653–10,219)
NCA results^*d*^	O. viverrini-infected individuals	9	1,398 (1,254–1,558)	8 (6–10)	4 (4–5)^*e*^	12,044 (11,055–13,910)^*f*^
Yuan et al.^*g*^^,^^*h*^	Healthy volunteers	12	640	4.20	4.74	4,450
Dou et al.^*g*^^,^^*i*^	Healthy volunteers	8	449	5.25	5.38	4,769
600 mg						
Compartmental model results	O. viverrini-infected individuals	9	953 (440–1,058)	6.54 (4.97–8.52)	5.00 (4.24–10.64)	10,831 (8,162–14,584)
NCA results^*d*^	O. viverrini-infected individuals	9	1,351 (1,294–1,561)	6 (4–8)	4 (4–5)^*e*^	14,003 (12,309–15,555)^*f*^
Yuan et al.^*g*^^,^^*h*^	Healthy volunteers	12	890	3.60	5.69	7,660
adADT						
25 mg						
Compartmental model results	O. viverrini-infected individuals	9	25 (5–34)	2.28 (2.06–2.43)	4.67 (3.70–5.00)	161 (44–235)
NCA results^*d*^	O. viverrini-infected individuals	9	26 (6–29)	4 (3–4)	5 (4–6)^*e*^	165 (48–239)^*f*^
50 mg						
Compartmental model results	O. viverrini-infected individuals	9	18 (16–42)	12.20 (6.18–14.20)	2.81 (2.26–3.58)	363 (199–398)
NCA results^*d*^	O. viverrini-infected individuals	9	53 (13–82)	6 (4–8)	4 (4–5)^*e*^	406 (196–655)^*f*^
100 mg						
Compartmental model results	O. viverrini-infected individuals	9	101 (91–109)	6.12 (4.42–8.47)	3.30 (2.51–3.67)	809 (690–1,013)
NCA results^*d*^	O. viverrini-infected individuals	9	117 (88–180)	5 (3–6)	4 (4–5)^*e*^	816 (715–1,043)^*f*^
200 mg						
Compartmental model results	O. viverrini-infected individuals	23	60 (38–237)	8.51 (6.04–9.79)	4.09 (3.17–4.61)	972 (436–2,399)
NCA results for 4 50-mg tablets^*d*^	O. viverrini-infected individuals	10	144 (46–243)	5 (4–7)	4 (4–5)^*e*^	2,057 (1,571–2,467)^*f*^
NCA results for 1 200-mg tablet^*d*^	O. viverrini-infected individuals	13	45 (34–142)	8 (6–10)	6 (5–8)^*e*^	574 (489–1,387)^*f*^
400 mg						
Compartmental model results	O. viverrini-infected individuals	9	116 (69–202)	9.22 (7.96–11.20)	4.83 (4.36–12.41)	2,049 (878–4,975)
NCA results^*d*^	O. viverrini-infected individuals	9	216 (56–398)	10 (8–10)	6 (5–7)^*e*^	1,654 (917–2,853)^*f*^
Dou et al.^*g*^^,^^*i*^	Healthy volunteers	8	148	7.13	7.09	1,989
600 mg						
Compartmental model results	O. viverrini-infected individuals	9	235 (215–387)	8.89 (5.52–11.90)	5.00 (4.24–10.63)	4,033 (2,958–6,050)
NCA results^*d*^	O. viverrini-infected individuals	9	345 (200–427)	8 (6–10)	6 (6–6)^*e*^	3,600 (2,456–4,571)^*f*^

a NCA, noncompartmental analysis (performed by using WinNonlin version 5.2; Pharsight Corporation, USA). Results are presented as medians (interquartile ranges) where available; it is not clear whether the results reported previously by Yuan et al. (15) and Dou et al. (13) are medians or means.

b Estimated by regression analysis of the terminal phase of model-predicted individual concentration-time profiles.

c AUC from 0 to 72 h for the compartmental model results and AUC from 0 h to infinity otherwise.

d Plasma results are shown, which are a subset of the data used in this study (27).

e Calculated where possible by using half-life = ln(2)/λ, where the elimination rate constant λ was determined by linear regression of the natural log-transformed concentration values in the elimination phase.

f Calculated where possible by using the trapezoidal rule of the natural log concentration-by-time profile.

g One-compartment model.

h See reference 15.

i See reference 13.

Previous PK studies of tribendimidine have been conducted only in healthy volunteers, who may be expected to have very different PK profiles (25). However, our estimates for *C*_max_ and half-life were broadly comparable to those reported in previous studies, which also used one-compartment models (Table 3) (8, 13). Our estimated *T*_max_ and AUC for dADT were typically higher than those reported previously but were comparable for adADT. We observed large variability in the estimated *T*_max_, except for the 25-mg dose, where split tablets were used. Since the enteric coating had been destroyed for the 25-mg dose, the absorption of the drug tended to be quicker and more consistent across patients. The long mean transit absorption time for dADT indicates slow absorption of the drug, with prolonged residence in the stomach. For doses of at least 100 mg, 94% of the estimated *C*_max_ values were above the 90% effective concentration (EC_90_) value of 75 ng/ml (27). Correspondingly, we observed relatively high cure rates of ≥55% with doses of at least 100 mg. There was a strong association between cure and both *C*_max_ and AUC values for dADT. There was no evidence of an association between cure and adADT exposure, confirming the marginal activity of this metabolite.

In conclusion, we have described for the first time a structural population PK model for tribendimidine in O. viverrini-infected individuals. Our findings will contribute to informing the incorporation of tribendimidine into the suite of drugs available for the treatment and control of helminthic infections. Further PK/PD studies of tribendimidine are needed to validate the structural model and confirm covariate relationships and associations between exposure parameters and cure.

## Supplementary Material

Supplemental material

## References

[1] Sripa B, Kaewkes S, Intapan PM, Maleewong W, Brindley PJ 2010 Food-borne trematodiases in Southeast Asia: epidemiology, pathology, clinical manifestation and control. Adv Parasitol 72:305–350. doi:10.1016/S0065-308X(10)72011-X. 20624536

[2] Van den Enden E 2009 Pharmacotherapy of helminth infection. Expert Opin Pharmacother 10:435–451. doi:10.1517/14656560902722463. 19191680

[3] Sayasone S, Odermatt P, Phoumindr N, Vongsaravane X, Sensombath V, Phetsouvanh R, Choulamany X, Strobel M 2007 Epidemiology of Opisthorchis viverrini in a rural district of southern Lao PDR. Trans R Soc Trop Med Hyg 101:40–47. doi:10.1016/j.trstmh.2006.02.018. 16828134

[4] Keiser J, Utzinger J 2010 The drugs we have and the drugs we need against major helminth infections. Adv Parasitol 73:197–230. doi:10.1016/S0065-308X(10)73008-6. 20627144

[5] Keiser J, Utzinger J 2005 Emerging foodborne trematodiasis. Emerg Infect Dis 11:1507–1514. doi:10.3201/eid1110.050614. 16318688 PMC3366753

[6] Panic G, Duthaler U, Speich B, Keiser J 2014 Repurposing drugs for the treatment and control of helminth infections. Int J Parasitol Drugs Drug Resist 4:185–200. doi:10.1016/j.ijpddr.2014.07.002. 25516827 PMC4266803

[7] Xiao S-H, Hui-Ming W, Tanner M, Utzinger J, Chong W 2005 Tribendimidine: a promising, safe and broad-spectrum anthelmintic agent from China. Acta Trop 94:1–14. doi:10.1016/j.actatropica.2005.01.013. 15777691

[8] Xiao S-H, Utzinger J, Tanner M, Keiser J, Xue J 2013 Advances with the Chinese anthelminthic drug tribendimidine in clinical trials and laboratory investigations. Acta Trop 126:115–126. doi:10.1016/j.actatropica.2013.01.009. 23352956

[9] Soukhathammavong P, Odermatt P, Sayasone S, Vonghachack Y, Vounatsou P, Hatz C, Akkhavong K, Keiser J 2011 Efficacy and safety of mefloquine, artesunate, mefloquine-artesunate, tribendimidine, and praziquantel in patients with Opisthorchis viverrini: a randomised, exploratory, open-label, phase 2 trial. Lancet Infect Dis 11:110–118. doi:10.1016/S1473-3099(10)70250-4. 21111681

[10] Yuan G, Xu J, Qu T, Wang B, Zhang R, Wei C, Guo R 2010 Metabolism and disposition of tribendimidine and its metabolites in healthy Chinese volunteers. Drugs R D 10:83–90. doi:10.2165/11539320-000000000-00000. 20698716 PMC3585841

[11] Yuan G, Wei C, Wang B, Zhang R, Xu J, Guo R 2008 Metabolism and excretion of tribendimidine in healthy human volunteers. Chin J New Drugs Clin Remedies 27:667–670. (In Chinese.)

[12] Xiao S, Xue J, Xu L, Zheng Q, Qiang H, Zhang Y 2009 The in vitro and in vivo effect of tribendimidine and its metabolites against Clonorchis sinensis. Parasitol Res 105:1497–1507. doi:10.1007/s00436-009-1579-6. 19655171

[13] Dou X, Yuan G, Zhang R, Wei C, Wang B, Guo R 2013 Pharmacokinetic study of dADT and acetylized dADT, metabolites of tribendimidine in human. J Pharm Res 32:543–545. (In Chinese.)

[14] Xu J, Yuan G, Wei C, Wang B, Guo R 2008 Determination of urinary tribendimidine metabolite-terephthalic acid by HPLC. J Shandong Univ Health Sci 46:1016–1019. (In Chinese.)

[15] Yuan G, Wang B, Wei C, Zhang R, Guo R 2008 LC-MS determination of p-(1-dimethylamino ethylimino)aniline: a metabolite of tribendimidine in human plasma. Chromatographia 68:139–142.

[16] Duthaler U, Keiser J, Huwyler J 2015 LC-MS/MS method for the determination of two metabolites of tribendimidine, deacylated amidantel and its acetylated metabolite in plasma, blood and dried blood spots. J Pharm Biomed Anal 105:163–173. doi:10.1016/j.jpba.2014.12.006. 25553533

[17] Beal S, Sheiner L, Boeckman A, Bauer R 1989–2009 NONMEM users' guides. Icon Development Solutions, Ellicott City, MD.

[18] Lindbom L, Ribbing J, Jonsson EN 2004 Perl-speaks-NONMEM (PsN)—a Perl module for NONMEM related programming. Comput Methods Programs Biomed 75:85–94. doi:10.1016/j.cmpb.2003.11.003. 15212851

[19] Tarning J, Thana P, Phyo AP, Lwin KM, Hanpithakpong W, Ashley EA, Day NPJ, Nosten F, White NJ 2014 Population pharmacokinetics and antimalarial pharmacodynamics of piperaquine in patients with Plasmodium vivax malaria in Thailand. CPT Pharmacometrics Syst Pharmacol 3:e132. doi:10.1038/psp.2014.29. 25163024 PMC4150927

[20] Beal SL 2001 Ways to fit a PK model with some data below the quantification limit. J Pharmacokinet Pharmacodyn 28:481–504. doi:10.1023/A:1012299115260. 11768292

[21] Holford NH 1996 A size standard for pharmacokinetics. Clin Pharmacokinet 30:329–332. 8743333 10.2165/00003088-199630050-00001

[22] Levey AS, Stevens LA, Schmid CH, Zhang YL, Castro AF III, Feldman HI, Kusek JW, Eggers P, Van Lente F, Greene T, Coresh J 2009 A new equation to estimate glomerular filtration rate. Ann Intern Med 150:604–612. doi:10.7326/0003-4819-150-9-200905050-00006. 19414839 PMC2763564

[23] Savic RM, Karlsson MO 2009 Importance of shrinkage in empirical Bayes estimates for diagnostics: problems and solutions. AAPS J 11:558–569. doi:10.1208/s12248-009-9133-0. 19649712 PMC2758126

[24] Greenblatt D, Koch-Weser J, Sellers EM, Shader RI 1982 Drug disposition in old age. N Engl J Med 306:1081–1088. doi:10.1056/NEJM198205063061804. 7040951

[25] Na Bangchang K, Karbwang J, Pungpak S, Radomyos B, Bunnag D 1993 Pharmacokinetics of praziquantel in patients with opisthorchiasis. Southeast Asian J Trop Med Public Health 24:717–723. 7939947

[26] Sayasone S, Odermatt P, Vonghachack Y, Xayavong S, Senggnam K, Duthaler U, Akkhavong K, Hattendorf J, Keiser J 26 7 2016 Efficacy and safety of tribendimidine against Opisthorchis viverrini: two randomised, parallel-group, single-blind, dose-ranging, phase 2 trials. Lancet Infect Dis. doi:10.1016/S1473-3099(16)30198-0. 27472949

[27] Duthaler U, Sayasone S, Vanobbergen F, Penny M, Odermatt P, Huwyler J, Keiser J 2016 Single-ascending-dose pharmacokinetic study of tribendimidine in Opisthorchis viverrini-infected patients. Antimicrob Agents Chemother 60:5705–5715. doi:10.1128/AAC.00992-16. 27431234 PMC5038241

