# Supplementary material for: Population Pharmacokinetic Modeling of Tribendimidine Metabolites in Opisthorchis viverrini-Infected Adults
Source: Antimicrob Agents Chemother. 2016 Sep 23;60(10):5695–704. doi: 10.1128/AAC.00655-16 (PMC5038332; doi:10.1128/AAC.00655-16)
Supplement: Supplemental material [file supp_60_10_5695__index.html]

Population Pharmacokinetic Modeling of Tribendimidine Metabolites in Opisthorchis viverrini-Infected Adults — Supplemental material 

# Population Pharmacokinetic Modeling of Tribendimidine Metabolites in Opisthorchis viverrini-Infected Adults

## Supplemental material

- Supplemental file 1 -

  Supplemental File S1: NONMEM code for final model.

  PDF, 220K
