# Supplementary material for: Population Pharmacokinetic Modeling of Tribendimidine Metabolites in Opisthorchis viverrini-Infected Adults
Source: Antimicrob Agents Chemother. 2016 Sep 23;60(10):5695–704. doi: 10.1128/AAC.00655-16 (PMC5038332; doi:10.1128/AAC.00655-16)
Supplement: Supplemental material [file AAC.00655-16_zac010165541so1.pdf]

Appendix

NONMEM code for final model

```
;;-----------------------------------------------------------;;
;; Modeller: Fiona Vanobberghen & Melissa Penny & Joel Tarning
;; Project name: Tribendimidine for Opisthorchis viverrini
;; Date: 2015-07-16
;;-----------------------------------------------------------;;
$PROBLEM    PK Tribendimidine
;-------------------------------------------
$INPUT      ID TIME DV AMT CMT MDV EVID BQL STUDY AGE WEIGHT FLUID CRCL
;-------------------------------------------
$DATA       data.xls
            IGNORE=@
;-------------------------------------------
$SUBROUTINE ADVAN6 TOL=5
;-------------------------------------------
$MODEL      COMP=(1)  ; Dose compartment
            COMP=(2)  ; Metabolite 1 (dADT) central compartment
            COMP=(3)  ; Metabolite 2 (AdADT) central compartment
            COMP=(4)  ; Transit compartment 1
            COMP=(5)  ; Transit compartment 2
            COMP=(6)  ; Transit compartment 3
            COMP=(7)  ; Transit compartment 4
            COMP=(8)  ; Transit compartment 5
            COMP=(9)  ; Transit compartment 6
;-------------------------------------------
$PK          ; MTSTUDY-DEFINITION START
             IF(STUDY.EQ.2) MTSTUDY = 1  ; Most common
             IF(STUDY.EQ.1) MTSTUDY = ( 1 + THETA(7))
             ; MTSTUDY-DEFINITION END

             ; MT-RELATION START
             MTCOV=MTSTUDY
             ; MT-RELATION END

             ; V2STUDY-DEFINITION START
             IF(STUDY.EQ.2) V2STUDY = 1  ; Most common
             IF(STUDY.EQ.1) V2STUDY = ( 1 + THETA(8))
             ; V2STUDY-DEFINITION END

             ; V2-RELATION START
             V2COV=V2STUDY
             ; V2-RELATION END

             ; V3STUDY-DEFINITION START
             IF(STUDY.EQ.2) V3STUDY = 1  ; Most common
             IF(STUDY.EQ.1) V3STUDY = ( 1 + THETA(9))
             ; V3STUDY-DEFINITION END

             ; V3-RELATION START
             V3COV=V3STUDY
             ; V3-RELATION END

             ; CMAGE-DEFINITION START
             CMAGE = ( 1 + THETA(10)*(AGE - 52.00))
             ; CMAGE-DEFINITION END
```

```
              ; CM-RELATION START
              CMCOV=CMAGE
              ; CM-RELATION END

              ; CPAGE-DEFINITION START
              CPAGE = ( 1 + THETA(6)*(AGE - 52.00))
              ; CPAGE-DEFINITION END

              ; CP-RELATION START
              CPCOV=CPAGE
              ; CP-RELATION END

              TVCP  = THETA(1)*((WEIGHT/51.50)**0.75)*CPCOV  ; Metabolite 1 CL
              CP    = TVCP*EXP(ETA(1))

              TVV2  = THETA(2)*((WEIGHT/51.50)**1.00)*V2COV  ; Metabolite 1 V
              V2    = TVV2*EXP(ETA(2))

              TVCM  = THETA(3)*((WEIGHT/51.50)**0.75)*CMCOV  ; Metabolite 2 CL
              CM    = TVCM*EXP(ETA(3))

              TVV3  = THETA(4)*((WEIGHT/51.50)**1.00)*V3COV  ; Metabolite 2 V
              V3    = TVV3*EXP(ETA(4))

              TVMT  = THETA(5)*MTCOV                         ; Mean transit time
              MT    = TVMT*EXP(ETA(5))

              S2    = V2                                     ; Scaling factor CMT2
              S3    = V3                                     ; Scaling factor CMT3

              NN    = 6
              KTR   = (NN+1)/MT
              K14   = KTR
              K45   = KTR
              K56   = KTR
              K67   = KTR
              K78   = KTR
              K89   = KTR
              K92   = KTR

              K20   = CP*0.35/V2        ; Renal elimination
              K23   = CP*0.65/V2        ; Hepatic elimination
              K30   = CM/V3             ; Hepatic elimination

;-------------------------------------------
$DES          DADT (1) = -A(1)*K14
              DADT (2) = A(9)*K92 - A(2)*K20 - A(2)*K23
              DADT (3) = A(2)*K23 - A(3)*K30
              DADT (4) = A(1)*K14 - A(4)*K45
              DADT (5) = A(4)*K45 - A(5)*K56
              DADT (6) = A(5)*K56 - A(6)*K67
              DADT (7) = A(6)*K67 - A(7)*K78
              DADT (8) = A(7)*K78 - A(8)*K89
              DADT (9) = A(8)*K89 - A(9)*K92
;-------------------------------------------
$ERROR        IF(CMT.EQ.2) IPRED = A(2)/S2
              IF(CMT.EQ.2) W     = SQRT(SIGMA(1,1))

              IF(CMT.EQ.3) IPRED = A(3)/S3
              IF(CMT.EQ.3) W     = SQRT(SIGMA(2,2))
```

```
              IF(IPRED.GT.0) IPRED = LOG(IPRED)

              ; lloq: was 1 ng/ml for plasma, blood and SECOND study DBS
              ; but was 10 ng/ml for FIRST study DBS
              IF(FLUID.EQ.1.OR.FLUID.EQ.2) LLOQ=1
              IF(FLUID.EQ.3.AND.STUDY.EQ.2) LLOQ=1
              IF(FLUID.EQ.3.AND.STUDY.EQ.1) LLOQ=10

              ; convert to nanonol/L
              IF(CMT.EQ.2) LLOQ = LLOQ*1000/173.214          ; dADT
              IF(CMT.EQ.3) LLOQ = LLOQ*1000/215.251          ; adADT

              ; take logs
              LLOQ = LOG(LLOQ)

              DUM  = (LLOQ-IPRED)/W
              CUMD = PHI(DUM)

              ; Prediction DV>=LOQ
              IRES  = IPRED-DV
              IWRES = IRES/W

              IF(BQL.EQ.0.AND.CMT.EQ.2) THEN
              F_FLAG = 0
              Y      = IPRED+ERR(1)
              ENDIF

              IF(BQL.EQ.0.AND.CMT.EQ.3) THEN
              F_FLAG = 0
              Y      = IPRED+ERR(2)
              ENDIF

              ; Likelihood DV<LOQ
              IF(BQL.EQ.1) THEN
              F_FLAG = 1
              Y      = CUMD + 0.000001
              ENDIF

;-------------------------------------------
$THETA        (0,16.8)                    ; 1.TVCL_MET1
              (0,112)                     ; 2.TVV2_MET1
              (0,42.4)                    ; 3.TVCL_MET2
              (0,30.4)                    ; 4.TVV3_MET2
              (0,2.16)                    ; 5.MTT
              (-0.066,-0.00987,0.071)     ; 6.CPAGE1
              (-1,1.05,5)                 ; 7.MTSTUDY1
              (-1,0.764,5)                ; 8.V2STUDY1
              (-1,0.743,5)                ; 9.V3STUDY1
              (-0.066,-0.0205,0.071)      ; 10.CMAGE1
;-------------------------------------------
$OMEGA        BLOCK(4)                    ; full var-cov matrix for CL and V
              0.0613
              0.171 0.662
              -0.129 -0.355 0.847
              0.0388 0.497 0.435 1.48
$OMEGA        0.486                       ; MTT
;-------------------------------------------
$SIGMA        0.621                       ; RUV dADT
              0.205                       ; RUV adADT
```

```
;------------------------------------------
$ESTIMATION   MAXEVAL=9999 POSTHOC METHOD=1 LAPLACIAN INTER
;------------------------------------------
$COVARIANCE   PRINT=E
;------------------------------------------
```
